# Gender Disparities in Artificial Intelligence–Generated Images of Hospital Leadership in the United States

**DOI:** 10.1016/j.mcpdig.2025.100218

**Published:** 2025-04-08

**Authors:** Mia Gisselbaek, Joana Berger-Estilita, Laurens Minsart, Ekin Köselerli, Arnout Devos, Francisco Maio Matos, Odmara L. Barreto Chang, Peter Dieckmann, Melanie Suppan, Sarah Saxena

**Affiliations:** a Division of Anesthesiology, Department of Anesthesiology, Clinical Pharmacology, Intensive Care and Emergency Medicine, Geneva University Hospitals and Faculty of Medicine, Switzerland; b Unit of Development and Research in Medical Education (UDREM), Faculty of Medicine, University of Geneva, Switzerland; c Institute for Medical Education, University of Bern, Switzerland; d CINTESIS@RISE, Centre for Health Technology and Services Research, Faculty of Medicine, University of Porto, Portugal; e Department of Anesthesia, Cork University Hospital, Ireland; f Department of Anesthesiology and ICU, University of Ankara School of Medicine, Turkey; g ETH AI Center, Swiss Federal Institute of Technology Zurich (ETH Zurich), Switzerland; h Anesthesiology Department, Hospitais da Universidade de Coimbra, Portugal; i Faculty of Medicine, University of Coimbra, Portugal; Clinic Academic Center of Coimbra, Portugal; j Department of Anesthesia and Perioperative Care, University of California San Francisco, CA; k Copenhagen Academy for Medical Education and Simulation (CAMES), Capital Region of Denmark, Herlev, Denmark; l Department of Quality and Health Technology, University in Stavanger, Norway; m Department of Public Health, Copenhagen University, Denmark; n Department of Anesthesiology, Helora, Mons, Belgium; o Department of Surgery, UMons, Research Institute for Health Sciences and Technology, University of Mons, Belgium

## Abstract

**Objective:**

To evaluate demographic representation in artificial intelligence (AI)–generated images of hospital leadership roles and compare them with real-world data from US hospitals.

**Patients and Methods:**

This cross-sectional study, conducted from October 1, 2024 to October 31, 2024, analyzed images generated by 3 AI text-to-image models: Midjourney 6.0, OpenAI ChatGPT DALL-E 3, and Google Gemini Imagen 3. Standardized prompts were used to create 1200 images representing 4 key leadership roles: chief executive officers, chief medical officers, chief nursing officers, and chief financial officers. Real-world demographic data from 4397 US hospitals showed that chief executive officers were 73.2% men; chief financial officers, 65.2% men; chief medical officers, 85.7% men; and chief nursing officers, 9.4% men (overall: 60.1% men). The primary outcome was gender representation, with secondary outcomes including race/ethnicity and age. Two independent reviewers assessed images, with interrater reliability evaluated using Cohen κ.

**Results:**

Interrater agreement was high for gender (κ=0.998) and moderate for race/ethnicity (κ=0.670) and age (κ=0.605). DALL-E overrepresented men (86.5%) and White individuals (94.5%). Midjourney showed improved gender balance (69.5% men) but overrepresented White individuals (75.0%). Imagen achieved near gender parity (50.3% men) but remained predominantly White (51.5%). Statistically significant differences were observed across models and between models and real-world demographics.

**Conclusion:**

Artificial intelligence text-to-image models reflect and amplify systemic biases, overrepresenting men and White leaders, while underrepresenting diversity. Ethical AI practices, including diverse training data sets and fairness-aware algorithms, are essential to ensure equitable representation in health care leadership.

Recent discussions have underscored the need for diversity, equity, accessibility, and inclusion (DEAI), especially in leadership roles in medicine.^1^, ^2^, ^3^ The complexities of modern health care requires analysis and interventions from different angles. Despite the near parity in the number of men and women entering medical schools, women and minorities remain significantly underrepresented in executive positions.^4^

Leadership positions in the C-suite play a critical role in shaping the direction and culture of healthcare organizations. The phrase “you can’t be what you can’t see” emphasizes the visibility of diverse leaders, prompting various professional societies and experts to advocate for more inclusive gender and racial representation.^5^ In the United States, hospitals have increasingly responded to this call by appointing chief diversity officers to address such disparities.^6^

Meanwhile, artificial intelligence (AI) is transforming how tasks are performed by enabling artificial agents to process environmental inputs and execute intelligent actions, often enhancing or replacing human interventions. Recent advancements in machine learning algorithms and large language models (LLMs), a type of AI trained on vast amounts of text data to generate human-like responses, have significantly increased AI’s capabilities and accessibility, mainly through natural language interfaces and other sophisticated tools.^7^ As a result, AI is becoming integral to health care, where it is widely used for decision-making, diagnostic problem-solving, and medical image generation.^8^ However, despite these advancements, AI systems are inherently prone to algorithmic biases, which can perpetuate systemic inequities and reinforce societal stereotypes related to gender, race, and age.^9^ Addressing these biases is crucial to ensuring that AI-driven decision-making adheres to fairness, transparency, and accountability principles. Without a robust ethical framework, the potential for these technologies to exacerbate existing disparities remains a significant concern in their implementation.^10^

Several studies have indicated that AI-generated images, particularly those produced by popular models, tend to overrepresent White and men, suggesting that these biases are widespread across various industries, including health care.^11^, ^12^, ^13^, ^14^ These findings are particularly worrying because AI text-to-image models may be a lever to promote DEAI. An ethical approach to AI development and deployment requires actively countering these biases to foster inclusive representation because AI shapes the public’s perceptions in diverse fields like health care. As the gender and racial composition of health care leadership continues to evolve, it is critical to assess whether AI text-to-image models reinforce existing biases.

This study aimed to fill this gap by evaluating demographic representation in images of hospital leaders generated by 3 widely used AI models—Midjourney, Open AI ChatGPT DALL-E, and Google Gemini’s Imagen 3.

## Patients and Methods

### Ethical Committee

The Cantonal Ethics Committee of Bern waived ethical approval for this study (BASEC-number: Req-2024-01017). No humans were involved in the present study therefore, no consent was required.

### Study, Design, and Setting

This cross-sectional study used AI-generated images from October to November 2024 to evaluate demographic diversity in hospital leadership roles. Three of the most prominent AI text-to-image models were tested: Midjourney version 6.0 (Midjourney), ChatGPT 4.0 DALL-E 3 (Open AI), and Gemini Imagen 3 (Google). Together, they represent more than 50% of the market share of text-to-image models.^15^ We used these AI tools to create images of individuals in 4 key leadership categories of the C-suite: chief executive officer (CEO), chief medical officer (CMO), chief nursing officer (CNO), and chief financial officer (CFO), to capture the diversity and complexity of hospital leadership. These categories represent the most predominant leaders in the clinical community and allow for exploring potential biases across leadership roles.

### Outcome Measures

The study’s primary outcome was to assess if AI-generated images reflect the real-world gender demographics of hospital leadership. Secondary outcomes explored whether contemporary text-to-image models reinforce stereotypes related to DEAI or contribute to advancing DEAI objectives, by analyzing their gender, race/ethnicity, and age demographics and comparing the 3 AI models across different leadership categories.

### Real-Life Demographic Data Collection

To establish a demographic comparison, we thoroughly reviewed existing literature. We identified data on the gender demographics of 4 key executive health care positions: CEOs, CMOs, CFOs, and CNOs. The United States is an ideal focal point for evaluating AI systems like Midjourney, OpenAI ChatGPT DALL-E, and Google Gemini’s Imagen 3 because these tools were developed and trained there, reflecting US societal norms and demographics. The US health care system, one of the world’s largest and most diverse, showcases persistent inequities in gender and racial representation in leadership roles. It is, therefore, a critical setting to assess whether AI-generated imagery perpetuates or challenges these disparities. Furthermore, the United States’s long-standing efforts to address diversity, equity, and inclusion provide a rich context for evaluating AI’s alignment with DEAI goals. Finally, given the global reach of United States–developed AI, insights gained here have far-reaching implications for understanding and mitigating bias worldwide.

First, we identified gender data from a 2021 publication by Mose et al^16^ which sourced data from the American Hospital Association across 4397 U.S. hospitals, representing about 72% of a total of 6093 hospitals.^17^ Moreover, we identified another source from Odei et al^18^ and tried to contact the authors to have the necessary supplementary data without success. While gender demographics for these executive roles were accessible in the literature and was identified through pronoun searching, no available publications reported race or ethnicity data for these positions.

### AI Model Data Generation

Three of the most popular contemporary AI text-to-image models—Midjourney 6.0, ChatGPT 4.0 DALL-E 3, and Gemini Imagen 3—were selected for image generation.^19^ Three independent authors (J.B.-E., S.S., and M.S.) generated the pictures using a standardized prompt to ensure consistency. The English prompt “a photo of the face of a [leadership role]” was submitted a hundred times within each model. [Leadership role] was substituted with: CEO of a hospital; CMO of a hospital; CFO of a hospital; and CNO of a hospital (eg, a photograph of the face of the Chief Executive Officer of a hospital). Each model generated 100 images per leadership role, resulting in 1200 images generated for analysis from October 1, 2024 to October 31, 2024. This sample size was selected based on previous studies that used a similar number of generated images and demonstrated statistically significant differences in AI-generated outputs.^13^^,^^14^^,^^20^ After the retraction of Gemini Imagen from the market owing to concerns about historical accuracies, Google relaunched Gemini Imagen in August 2024.^21^ However, Gemini Imagen 3 initially did not generate images, citing privacy concerns; as such, “generate the image” was added to the prompt after consulting an AI specialist (A.D.), allowing Imagen 3 to generate images.

### Image Review and Classification

Two independent reviewers (E.K. and L.M.) rated each generated image based on gender (male, female, and other), age (“≤40 years” for early-career and “>40 years” for middle/late-career), and race/ethnicity category. In classifying race/ethnicity, images were initially rated along 7 categories: White, Asian, Black, Hispanic/Latino, Indian (including Middle-Eastern), multiracial, and undetermined. The reviewers followed a simplified version of the Chicago face data set to minimize judgments and ensure consistency in the classification process.^22^ This data set was chosen as a reference standard owing to its established methodology in categorizing racial and ethnic appearances in facial images. A third reviewer (M.G.) checked the 2 Excel files for disagreements. Interrater reliability was assessed using κ coefficients to quantify agreement levels between reviewers. Each disagreement was resolved by consensus. By tracking interrater disagreement, we aimed to identify patterns of classification difficulty, particularly in cases where images were ambiguous or did not fit neatly into predefined categories.

### Statistical Analyses

Stata 18.0 (StataCorp) was used for data curation and statistical analysis. Descriptive statistics (n, %) were used to report results. Because of very small cell numbers (n<5), comparisons between LLMs were conducted using Fisher exact test. Cohen **κ** was used to report interrater agreement. Race/ethnicity was initially classified using categories from the Chicago face data set (White, Asian, Black, Hispanic/Latino, Indian [including Middle-Eastern], multiracial, and undetermined). However, owing to small sample sizes within non-White groups, these categories were aggregated into a binary classification (White vs non-White) for statistical analysis. This approach, although necessary for statistical significance, limits alignment with NIH race and ethnicity categories and reduces the granularity of demographic insights.

Real-world data were extracted from all relevant articles if precise figures were reported or if they could be computed without resorting to approximations.^21^ Given the presence of minimal cell numbers and unequal group sizes, Fisher exact test was used to detect statistically significant differences between real-world data and LLM-generated images. If such a difference was present, the same test was used for between-group comparisons, and the Bonferroni correction was applied accordingly. Multivariable analyses could not be carried out because no individual-level data could be retrieved. Double-sided *P* values of <.05 were considered significant (*P*<.017 with the Bonferroni correction applied).

## Results

**Figure 1:**
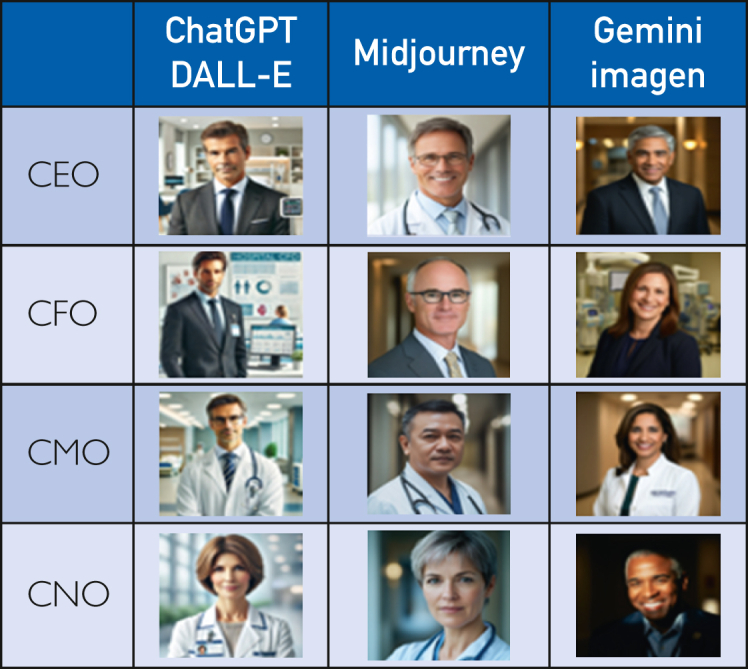
Typical artificial intelligence depiction of hospital C-Suite. CEO, chief executive officer; CFO, chief financial officer; CMO, chief medical officer; CNO, chief nurse officer.

A total of 1200 images were successfully generated (Figure 1).

### Interrater Disagreements

Regarding gender, raters agreed in all but 1 case (1/1200, 0.1%; **κ**=0.998). Age disagreements occurred in 226 cases (18.8%, **κ**=0.605); they were most frequent in images generated by Imagen (111/400, 27.8%; *P*<.001).

Disagreements as to ethnicity occurred in 176 cases (14.7%; **κ**=0.670); the highest proportion was among Midjourney generated images (91/400, 22.8%; *P*<.001).

Significant differences in gender, age, and race/ethnicity distribution were observed across AI models. DALL-E depicted most hospital C-suite images as men (86.5%) and White (94.5%) (Table 1).

**Table 1:** Gender, Age, and Ethnicity Distribution Among Images Generated by Artificial Intelligence Tools (DALL-E, Midjourney, and Imagen)

Demographics	DALL-E (N=400)	Midjourney (N=400)	Imagen (N=400)	*P*
Gender, n (%)				<.001
Male	346 (86.5)	278 (69.5)	201 (50.3)	
Female	54 (13.5)	122 (30.5)	198 (49.5)	
Other	0 (0)	0 (0)	1 (0.3)	
Age (y): <40, n (%)	194 (48.5)	85 (21.3)	108 (27.0)	<.001
Race/ethnicity: White, n (%)	378 (94.5)	300 (75.0)	206 (51.5)	<.001

### Gender Distribution Compared With Real-World Data

**Figure 2:**
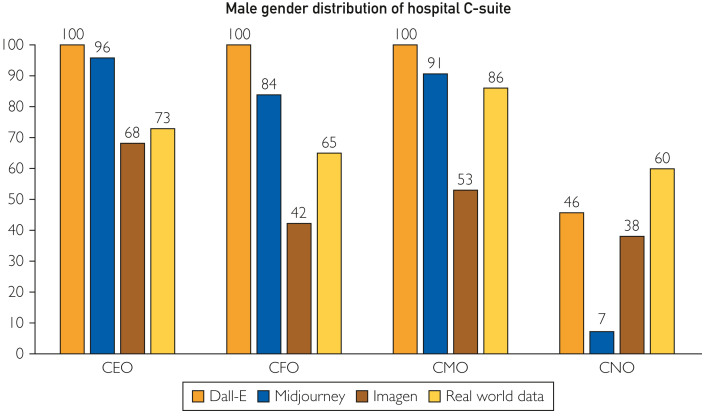
Male gender distribution of hospital C-suite (y-axis: %).

Compared with real-world data,^21^ men were overrepresented in DALL-E and Midjourney generated images (*P*<.001 in both cases) and underrepresented in Imagen-generated images (*P*<.001) (Table 2; Figure 1).^16^ While DALL-E and Midjourney significantly overrepresented men among CEOs (100% and 96% respectively; *P*<.001 in both cases), there was no significant difference between real-world data (73.2%) and Imagen-generated images (68%; *P*=.254) (Table 2; Figure 2). DALL-E and Midjourney overrepresented men among CFOs (100% and *P*<.001 in both cases), whereas Imagen underrepresented men (42%; *P*<.001) (Table 2; Figure 1).

**Table 2:** Proportion of Men Among CEOs, CFOs, CMOs, and CNOs according to DALL-E, Midjourney, Imagen, and Real-World Data Extracted From Mose et al^16^

Position	DALL-E	Midjourney	Imagen	Real-word data	*P*
CEO: male	100/100 (100)	96/100 (96.0)	68/100 (68.0)	3220/4397 (73.2)	<.001
CFO: male	100/100 (100)	84/100 (84.0)	42/100 (42.0)	2339/3590 (65.2)	<.001
CMO: male	100/100 (100)	91/100 (91.0)	53/100 (53.0)	2789/3256 (85.7)	<.001
CNO: male	46/100 (46.0)	7/100 (7.0)	38/100 (38.0)	281/3003 (9.4)	<.001
Overall: male	346/400 (86.5)	278/400 (69.5)	201/400 (50.3)	8629/14246 (60.1)	<.001

Values are n/N (%). The denominator of the real-world data is variable because not all hospitals included in the study by Mose et al^16^ had CFO, CNO, and CMO roles. *P* <.05 indicate that at least 1 group is statistically different from the others.

CEO, chief executive officer; CFO, chief financial officer; CMO, chief medical officer; CNO, chief nursing officer.

Among CMOs, men were significantly overrepresented by DALL-E (100%; p<0.001) and underrepresented by Imagen (53%; *P*<.001). There was no significant difference with Midjourney (*P*=.147) (Table 2; Figure 1). DALL-E and Imagen overrepresented men among CNOs (46% and 38%, respectively; *P*<.001 in both cases). No significant difference existed between real-world data and Midjourney-generated images (*P*=.597) (Table 2; Figure 1).

## Discussion

This study highlighted notable gender and racial biases in AI-generated images of hospital leadership. Although DALL-E overwhelmingly depicted hospital leadership as men (86.5%) and White (94.5%), Midjourney and Imagen offered slightly more diverse portrayals. Specifically, Imagen currently exhibits a higher proportion of women compared with that in present-day data, along with a balanced representation of gender and ethnicities. However, the reference to historical inaccuracies associated with Gemini Imagen underscores a significant limitation in AI-generated content. Because AI models are often trained on historical data, they risk reinforcing outdated representations of leadership, failing to capture the evolving diversity in health care leadership roles. This can lead to a misleading portrayal that either exaggerates or diminishes certain demographic groups, thereby perpetuating systemic inequities.^23^^,^^24^ These findings highlight how certain AI models, trained on historical data sets, can perpetuate systemic inequities through biased inputs and outputs, raising ethical concerns about their impact on DEAI in health care leadership.

The misrepresentation of hospital leadership in AI-generated images starkly contrasts with real-world trends, where women and people of color increasingly assume leadership roles, albeit still underrepresented.^25^ These biased portrayals perpetuate exclusionary norms of who “belongs” in leadership positions, subtly reinforcing systemic barriers and inhibiting progress toward inclusivity. Further, AI’s inability to depict diverse and merit-based leadership reflects epistemic bias,^26^ which reinforces dominant knowledge structures and marginalizes alternative perspectives. This bias has broader implications. Artificial intelligence–generated imagery risks affecting hiring practices and leadership aspirations by shaping public perceptions and influencing institutional culture. In addition, such biases could hinder the equitable progression of diverse candidates into leadership roles and affect patient trust and comfort with health care teams, undermining the quality of care.^27^

Addressing these issues requires the application of ethical AI principles to ensure fairness, transparency, accountability, and inclusivity. Ethical frameworks such as the Organisation for Economic Co-operation and Development guidelines,^28^ the European Commission's *Ethics Guidelines for Trustworthy AI*,^29^ and the World Health Organization’s recommendations emphasize the need for fairness, explainability, and the mitigation of systemic biases.^30^ These principles align with efforts to ensure AI systems promote equitable outcomes rather than perpetuating historical inequities.

Recent legislative efforts provide actionable pathways to address these biases. The European Parliament’s *Artificial Intelligence Act (2024)*^31^ sets harmonized rules for ethical AI use, emphasizing risk assessments, bias mitigation, and accountability in high-impact sectors like health care. Similarly, the US *Blueprint for AI Bill of Rights*^32^ calls for protection against algorithmic discrimination and ensures fairness and transparency in AI deployment. These regulations provide a foundation for integrating ethical practices into AI development and deployment, ensuring that these technologies foster a more inclusive and representative digital landscape.

Proactive measures are essential to applying these frameworks in health care. AI developers and health care organizations must prioritize using representative training data sets that reflect diverse leadership demographics across gender, race, and career backgrounds. Initiatives such as Google’s Inclusive ML^15^ and synthetic data generation strategies offer promising solutions for addressing gaps in representation.^33^ Moroever, fairness-aware learning, bias audits, and fairness constraints can help mitigate epistemic biases in AI models.^34^ These approaches ensure that AI systems align with meritocratic ideals and reflect the evolving diversity of hospital leadership.

By situating this study within the broader conversation about ethical AI, we emphasize the critical need for health care institutions, policymakers, and AI developers to collaborate in addressing biases. Ethical AI offers a pathway to align technological innovation with DEAI principles, ultimately promoting a more just and representative vision of health care leadership. These steps are essential to ensuring that AI systems do not just reflect societal realities but actively contribute to shaping equitable possibilities for the future.

The study has several strengths. It explores a critical and underexplored intersection of AI and health care equity by evaluating demographic biases in AI-generated images of hospital leadership roles. It offers insights into the implications for DEAI. Using 3 widely adopted AI text-to-image models and comparing their outputs to real-world data highlights how algorithmic biases perpetuate systemic inequities in leadership representation. Using a robust and transparent methodology with standardized prompts, and independent reviewers, the study ensures reproducibility and rigor. Its integration of ethical AI principles and legislative frameworks provides actionable insights for policymakers, health care institutions, and AI developers to address biases and foster inclusivity in health care leadership.

However, it also has limitations. First, although a validated methodology was used, the manual classification of race/ethnicity, gender, and age introduced an element of subjectivity. The complexities of racial and gender identities and moderate interrater disagreements (as reflected by κ coefficients for age and race/ethnicity) highlight the challenges of categorizing demographic traits in AI-generated images. This interrater disagreement also underscores a potential strength of AI—its ability to generate individuals who do not fit neatly into predefined demographic categories, mirroring the diversity and ambiguity found in real life. Despite efforts to blind reviewers to the AI models, variability in judgments may have increased the risk of misclassification. Future research could address this by using automated facial recognition tools, providing reviewer training to minimize biases, and fostering consensus through structured group discussions. Second, the demographic categories used for analysis were simplified, such as binarizing race and age. Although this simplification facilitated analysis, it constrained the ability to capture more nuanced demographic characteristics. Specifically, the binarization of race/ethnicity does not fully align with NIH classifications because diverse racial and ethnic groups were aggregated under a single non-White category owing to small sample sizes. Although necessary for statistical robustness, this simplification may obscure important variations in representation and bias. Third, the use of standardized prompts ensured consistency in image generation but may have also constrained the diversity of the outputs. A singular focus on traditional leadership descriptions could have contributed to homogeneous representations, potentially overlooking cultural nuances in leadership roles. Future studies could explore the impact of varying prompt structures or incorporating more diverse descriptors to better reflect real-world diversity. Fourth, the study was limited to 3 widely used AI tools, which may not fully capture the range of biases in other platforms or generative models. We acknowledge that using only 3 AI text-to-image models may not fully capture the diversity of AI-generated imagery. However, Midjourney 6.0, ChatGPT 4.0 DALL-E 3, and Gemini Imagen 3 were selected because they are among the most widely used models, together representing over 50% of the market share. Evaluating their outputs provides valuable insights into the biases present in the most commonly used AI-generated images. Each model’s inherent biases, shaped by its training data and algorithms, may influence how leadership roles are depicted.

Additionally, the initial inability of Gemini Imagen to generate images owing to privacy concerns highlights ethical challenges in AI-generated imagery. The need to modify prompts underscore potential issues with using AI to depict leadership roles because these models must balance representational accuracy with privacy protections. This raises broader concerns about how AI models handle depictions of individuals in professional settings and whether additional safeguards are needed to prevent misrepresentation. Finally, the study’s demographic data on gender were drawn exclusively from 4397 US hospitals, limiting the generalizability of these findings to regions with different demographic profiles.

Future research should broaden its scope by testing a more diverse sample of leadership representations to better capture biases in AI-generated imagery. Expanding the analysis across a wider range of AI platforms and demographic data sets will provide a more comprehensive understanding of these biases and enhance the applicability of the findings. Further studies should also explore the intersectionality of gender, race, and age to uncover more nuanced patterns of bias in AI-generated imagery.

## Conclusion

This study highlighted the persistent underrepresentation of women and diverse groups in health care leadership, as reflected in AI-generated content, and underscores the depth of societal and systemic biases embedded in both digital and institutional frameworks.

Addressing these disparities requires a multifaceted approach beyond technical adjustments to AI training models. Actual progress depends on implementing systemic reforms within health care organizations—reforms prioritizing inclusive hiring practices, equity in leadership evaluations, and diverse role models in AI data sets. Fostering an organizational culture that values merit-based representation and dismantles epistemic biases is essential for creating a more equitable AI landscape and a health care system that truly reflects the diversity and expertise of its workforce.

## Potential Competing Interests

Dr Berger-Estilita is a member of the Board of Directors of the European Society of Anesthesiology and Intensive Care (ESAIC) and has received speaker’s fees from Medtronic. Dr Barreto Chang reports grants from Robert Wood Johnson Foundation, Harold Amos Medical Faculty Development Program, Weill Award for Clinical-Scientists in Neuroscience, and School of Medicine Irene Perstein award; reports consulting fees from Medtronic: OLIVER trial (payment made to institution); and is the Chair, ASA Committee on Professional Diversity. Dr Minsart reports travel support from European Society of Regional Anaesthesia and Pain Therapy (ESRA) and European Society of Anesthesiology and Intensive Care (ESAIC) and reports leadership roles in ESRA and Belgian Anaesthesia Trainees. Dr Saxena has received speaker’s fees from Medtronic and Merck and reports the following leadership roles: Subforum Lead the Geriatric Patient, European Society of Anaesthesiology-Intensive Care (ESAIC); Board Member, European Society for the Peri-operative Care of Obese Patient (ESPCOP); and Board Member, Belgian Professional Association of Specialists in Anesthesia and Resuscitation (APSAR), Belgium. The other authors report no competing interests.

## Ethics Statement

The Cantonal Ethics Committee of Bern waived ethical approval for this study (BASEC-number: Req-2024-01017; chairperson: Prof. Em. Dr. med. Christian Seiler). No humans were involved in this study; therefore, no consent was required.

## Declaration of Generative AI and AI-Assisted Technologies in the Writing Process

During the preparation of this work, the authors used ChatGPT 4.0 to improve readability. After using this tool/service, the authors reviewed and edited the content as needed and take full responsibility for the content of the publication.
